# To have or not to have: expression of amino acid transporters during pathogen infection

**DOI:** 10.1007/s11103-022-01244-1

**Published:** 2022-02-01

**Authors:** Laura Tünnermann, Justine Colou, Torgny Näsholm, Regina Gratz

**Affiliations:** 1 Department of Forest Genetics and Plant Physiology, Umeå Plant Science Centre, Swedish University of Agricultural Sciences, 90183 Umeå, Sweden; 2 Department of Forest Ecology and Management, Swedish University of Agricultural Sciences, 90183 Umeå, Sweden

**Keywords:** Amino acids, Amino acid transporter, Lysine histidine transporter (LHT), Organic nitrogen, Pathogen defense, Ethylene signaling

## Abstract

The interaction between plants and plant pathogens can have significant effects on ecosystem performance. For their growth and development, both bionts rely on amino acids. While amino acids are key transport forms of nitrogen and can be directly absorbed from the soil through specific root amino acid transporters, various pathogenic microbes can invade plant tissues to feed on different plant amino acid pools. In parallel, plants may initiate an immune response program to restrict this invasion, employing various amino acid transporters to modify the amino acid pool at the site of pathogen attack. The interaction between pathogens and plants is sophisticated and responses are dynamic. Both avail themselves of multiple tools to increase their chance of survival. In this review, we highlight the role of amino acid transporters during pathogen infection. Having control over the expression of those transporters can be decisive for the fate of both bionts but the underlying mechanism that regulates the expression of amino acid transporters is not understood to date. We provide an overview of the regulation of a variety of amino acid transporters, depending on interaction with biotrophic, hemibiotrophic or necrotrophic pathogens. In addition, we aim to highlight the interplay of different physiological processes on amino acid transporter regulation during pathogen attack and chose the LYSINE HISTIDINE TRANSPORTER1 (LHT1) as an example.

**Supplementary Information:**

The online version contains supplementary material available at 10.1007/s11103-022-01244-1.

## Availability of nitrogen orchestrates plant pathogen resistance

A proper plant nitrogen (N) nutrition is warranted by the uptake of inorganic and organic N sources. Organic N such as proteins, peptides or amino acids (AAs) are taken up via specific root transporters (Paungfoo-Lonhienne et al. 2008; Nasholm et al. 2009; Tegeder and Rentsch 2010; Inselsbacher and Näsholm 2012; Tegeder and Masclaux-Daubresse 2018; Gratz et al. 2021) that have multiple functions within a plant (Yang et al. 2020; Yao et al. 2020). AAs represent an important storage and transport form of organic N and are precursors for protein synthesis. AAs are especially important for the development of roots, leaves, and seeds (Rentsch et al. 2007; Tegeder and Masclaux-Daubresse 2018), which makes AA transport systems a key component for plant development. Not only plants but also the microbial community relies on the availability of AAs, and it is not surprising that both compete for this N source (Roberts and Jones 2012; Kuzyakov and Xu 2013; Wilkinson et al. 2014). We identified the need of a concise survey highlighting the role of AA transporters (AATs) during pathogen infection due to the fact that literature mostly focusses on the influence of inorganic N on plant resistance (Ballini et al. 2013; Huang et al. 2017; Farjad et al. 2018; Sun et al. 2020).

Amino acid pools and fluxes are, however, dependent on N supply and the absolute majority of studies reporting on N effects on pathogen resistance have focused on comparisons of the inorganic N sources nitrate (NO_3_^−^) and ammonium (NH_4_^+^). In addition to reviewing the links between AATs and pathogen resistance, we therefore also performed a literature search aiming to compare effects of nitrate and ammonium addition on the plant’s ability to resist pathogens that differ in their nutrition strategy (Table 1, Supplementary Tables 1–3). Especially the different nutrient acquisition strategies by different pathogens such as biotrophic, hemibiotrophic as well as necrotrophic pathogens are important in this context. Biotrophic pathogens exhibit specialized feeding structures that allow nutrient retrieval from living cells. Hemibiotrophic microbes, however, first colonize the living cell but then transition into a necrotrophic phase. Necrotrophs obtain their nutrients from killed cells (Spanu and Panstruga 2017). Within biotrophic pathogens, the presence of different inorganic N sources led to strong and opposing effects: addition of NO_3_^−^ reduced plant resistance in the majority of analyzed cases (11 out of 15 cases). Interestingly, not only the presence but also the rate of NO_3_^−^ addition influenced defense responses of plants (Ding et al. 2021). Tomato plants infected with the biotroph *Ralstonia solanacearum*, for instance, demonstrated less disease lesions when grown on 1 mM compared to 7 mM NO_3_^−^ (Ding et al. 2021). Interestingly, the presence of NH_4_^+^ as N source, though, demonstrated an opposing trend: in 10 out of 14 cases elevated plant resistance was found (Table 1, Supplementary Table 1). Plant resistance against hemibiotrophic pathogens seems not to display any clear response to different inorganic N sources and both positive and negative effects of NO_3_^−^ and NH_4_^+^ addition have been reported (Table 1, Supplementary Table 2). Concentration-related effects such as reduced disease lesions were observed for tomato plants after infection with *Pseudomonas syringae* (*P. syringae*), when plants were grown on 1 mM compared to 7 mM NO_3_^−^ (Ding et al. 2021). In 9 out of 15 cases NO_3_^−^ led to a positive immune response such as increased resistance or hypersensitive response during necrotrophic attack (Table 1, Supplementary Table 3). Similar to biotrophic and hemibiotrophic infection, plant responses after necrotrophic interaction seem to dependent on the N rate (Farjad et al. 2018). Measurements of bacterial cell numbers of the necrotroph *Erwinia amylovora* in infected *Arabidopsis thaliana* (*Arabidopsis*) revealed lower numbers when grown on low NO_3_^−^ (0.5 mM) compared to high NO_3_^−^ (5 mM). This was associated with transcriptional reprograming of defense genes, e.g., *PATHOGENESIS-RELATED GENE*2 and 5 (*PR2* and *PR5*) or salicylic acid (SA)-related genes (Farjad et al. 2018). Addition of NH_4_^+^, though, led to increased cases of elevated plant susceptibility, when infected with a necrotroph (7 out of 11 cases) (Table 1). Overall, we found that a plant’s ability to withstand biotrophic attacks tends to be more successful when NH_4_^+^ is accessible, the opposite of what was shown for necrotrophs. The overall N addition rate might serve as a proxy for plant N status, which influences susceptibility additionally.

**Table 1:** Effects of nitrate (NO_3_^−^) and ammonium (NH_4_^+^) availability on plant pathogen resistance

Type	Nutrition strategy	Positive effect of NO_3_^−^ on plant resistance	Negative effect of NO_3_^−^ on plant resistance	Positive effect of NH_4_^+^ on plant resistance	Negative effect of NH_4_^+^ on plant resistance
Bacteria	Biotroph	1	3	1	0
Fungi	Biotroph	1	4	2	1
Nematode/Protist	Biotroph	1	3	3	1
Virus	Biotroph	0	1	4	1
Oomycota	Biotroph	1	0	0	1
Bacteria	Hemibiotroph	1	2	2	1
Fungi	Hemibiotroph	5	3	3	5
Oomycota	Hemibiotroph	1	1	1	1
Bacteria	Necrotroph	0	2	0	0
Fungi	Necrotroph	9	4	4	7
**Total**	**Biotroph**	**4**	**11**	**10**	**4**
	**Hemibiotroph**	**7**	**6**	**6**	**7**
	**Necrotroph**	**9**	**6**	**4**	**7**

Results of a survey of different studies are summarized, comparing different pathogen types, separated by their nutrition strategy. The impact of different inorganic N sources on the plant’s immune response during respective pathogen attacks were denoted. Effects are expressed through increased resistance and elevated susceptibility, respectively. Respective numbers express the count of experiments found, displaying a similar response. A summary of the counts is presented in bold, with no differentiation between different pathogen types, but grouped according to nutrition strategy. Respective references to the included studies can be found in Supplementary Tables 1–3

As shown above, plant N sources play critical roles for plant resistance. This observation motivates a further analysis of N transporters during pathogen attack. Camanes et al. (2012) investigated the response of NO_3_^−^ transporters AtNRT2.1 and AtNRT2.2 to infection by the hemibiotrophic bacteria *P. syringae*. The *nrt2* mutant exhibited an increased immune response along with a reduced susceptibility and significant alterations in the transcriptome. The expression of SA marker genes was strongly increased compared to the wild type, and it was suggested that members of the AtNRT2 family might be important for the plant-pathogen interaction (Camanes et al. 2012). More recently it was shown that the *nrt2.5* mutant displayed similar responses (du Toit et al. 2020). Similarly, also NH_4_^+^ transporters such as AtAMT1.1 seem to play an important role for plant resistance (Pastor et al. 2014). *amt1.1* plants infected with *P. syringae* and *Plectosphaerella cucumerina*, a hemibiotrophic and a necrotrophic organism respectively, exhibited increased resistance, an effect that was enhanced by N depletion (Pastor et al. 2014). These findings lead to the hypothesis, that N transporters play a role in plant immune responses, by acting as regulators in N supply. We therefore ask the question whether other transporters that are involved in N uptake and N translocation and in particular the AATs could potentially also play a role in plant resistance.

## A dual utilization of amino acids

It is well established that pathogens can feed on plant N reserves, mainly AAs, which makes them crucial players in the plant-pathogen interaction (Struck et al. 2004; Zeier 2013; Sonawala et al. 2018; Yang et al. 2020; Sharma 2020). It is energetically more beneficial for pathogens to directly acquire and metabolize plant AAs which is why a range of pathogens can directly target the induction of genes needed for AAT (Sonawala et al. 2018; Li et al. 2020). Having control over a plant’s AA uptake and transport system can, therefore, be decisive for the survival of either the plant or the pathogen.

Li et al. found substantial reprogramming of N and C metabolic pathways in kiwifruit tissues upon infection with *P. syringae*, i.e., an accumulation of specific AAs (Li et al. 2020). While the accumulation of some AAs can be beneficial for the pathogen, others can play important roles in plant resistance. Tryptophan and methionine, for instance, are known precursors for the synthesis of secondary metabolites with antimicrobial effects (Ahuja et al. 2012). Depending on the microbe, these metabolites accumulate in individual root cell layers and can contribute to increased resistance (Froschel et al. 2021). A similar response of citrus plants was described upon infection with the phloem-feeding biotroph *Candidatus liberibacter*, as the phloem sap of tolerant plants exhibited high amounts of tryptophan, tyrosine or phenylalanine; well-studied precursors for secondary metabolites and phenolics (Killiny and Hijaz 2016). Proline, a known radical scavenger, contributes to the regulation of cellular redox homeostasis (Smirnoff and Cumbes 1989). Gupta et al. (2020) recently corroborated the positive properties of proline during infection and analyzed upstream components. They identified miRNA involved in the regulation of proline biosynthesis, which is not only important for the plant immune response but is also involved in regulation of abiotic stresses (Gupta et al. 2020).

The above suggests that it is crucial to understand the molecular regulation of AA transport and accumulation because AAs can be used as N sources for the pathogen but also as protective agents for the plant. This leads to the question whether AATs are differently expressed during plant-pathogen interaction and if so, who the driver of this regulation is. Having control over the expression can, thus, decide over the fate of both, plants or pathogens (Hammes et al. 2006; Liu et al. 2010; Elashry et al. 2013; Pariyar et al. 2018; Sonawala et al. 2018; Froschel et al. 2021).

## Responses of plant amino acid transporters to pathogen infection

The products of about 100 genes are known to facilitate AA transport in *Arabidopsis* and similar AATs have additionally been identified in many crop and tree species (Tegeder and Ward 2012; Pratelli and Pilot 2014; Yang et al. 2020). ATF (amino acid transporter family), APC (amino acid-polyamine-choline transporter family) and UMAMIT (usually multiple acids move in and out transporter family) represent the three main AAT families (Rentsch et al. 2007; Pratelli and Pilot 2014; Dinkeloo et al. 2018; Yang et al. 2020). ATFs can be divided into several subfamilies such as, e.g., AAPs (amino acid permeases) or LHTs (lysine histidine transporters) (Rentsch et al. 2007). CATs (cationic amino acid transporters) represent a subfamily within the APCs (Tegeder and Rentsch 2010).

### Amino acid permeases (AAPs)

AAPs, a group of one-directional transporters, are involved in root AA uptake, phloem loading, xylem-phloem transfer, and seed loading (Fischer et al. 1995; Okumoto et al. 2002, 2004; Lee et al. 2007; Svennerstam et al. 2008; Zhang et al. 2010; Santiago and Tegeder 2016). It is well known that AAPs are highly conserved between various species (Benedito et al. 2010; Zhao et al. 2012, 2017; Limpens et al. 2013; Garneau et al. 2018; Duan et al. 2020; Llebrés et al. 2021; Omari Alzahrani 2021).

Several members of the AAP family were found to be differentially regulated upon biotrophic interactions. AtAAPs demonstrated enhanced gene expression after plant-parasitic nematode infection and increased resistance in respective knockout mutants (Hammes et al. 2005; Elashry et al. 2013; Marella et al. 2013). Analysis of *aap1*, *aap2* and *aap6* knockout mutants displayed decreased reproduction of cyst nematodes (Elashry et al. 2013). Similarly, *aap3* and *aap6* exhibited reduced reproduction of root-knot nematodes (Marella et al. 2013). Recently, the role of CsAAP2A in cucumber became evident as knockout plants displayed resistance to downy mildew (Berg et al. 2021). A functional analysis of AAPs in tomato plants, when challenged with the hemibiotrophic *Phytophthora infestans* (*P. infestans*), displayed that mutations in the tomato homologues *SIAAP5A* and *SIAAP5B* led to similar effects (Berg et al. 2021). It is reasonable that an infection causes a differential regulation of local AATs in specific cell types. It would also be conceivable that a transporter is being regulated in opposing directions upon infection of the same pathogen, however, in different cells. A recent study zoomed in on these questions and compared expression patterns in four specific root cell layers (rhizodermis, cortex, endodermis, and stele), when *Arabidopsis* was challenged with, in their nutrition strategy varying, microbes (Froschel et al. 2021). When looking at the cell layer-specific transcript abundance after hemibiotrophic *P. parasitica* infection, it was found that *AtAAP3*, *AtAAP5* and *AtAAP6* were induced in the stele, however, *AtAAP6* was additionally upregulated in the cortex (Froschel et al. 2021). Responses to hemibiotrophic, vascular *Verticillium longisporum* (*V. longisporum*) varied within the AtAAP family: *AtAAP4* was the only representative that was upregulated and only in the cortex. *AtAAP1*, in the cortex, and *AtAAP2*, in the rhizodermis, were found to be downregulated after infection (Froschel et al. 2021).

Based on the above publications, it can be suggested that AAPs are negative regulators in plant defense against (hemi-) biotrophic pathogens. An increase in AAT transcript abundance might reduce plant defense reactions which would be beneficial for the pathogen. Alternatively, these transporters might be exploited by pathogens to steer plant AA transport, elevating the amount of accessible AAs in infected leaves and creating an artificial sink that pathogens can feed on (Berg et al. 2021).

### Cationic amino acid transporters (CATs)

Some AATs affect the plant immune system in a positive way, like AtCAT1 (Yang et al. 2014). The infection with hemibiotrophic *P. syringae* caused elevated transcript levels of *AtCAT1* and increased resistance. Overexpression of *AtCAT1* led to the constitutive expression of SA related and *PR1* genes, as well as an increase in SA levels. Since *AtCAT1* expression responded quickly to the infection it seems that it is involved in the systemic resistance of the plant (Yang et al. 2014).

### Usually multiple acids move in and out transporter family (UMAMITs)

Most AATs operate as one-directional symporter, transporting AAs along a proton gradient (Bush 1993; Frommer et al. 1993; Hsu et al. 1993), however, UMAMITs are an exception. Driven by an electrochemical gradient, UMAMITs transport AAs in both directions (Ladwig et al. 2012; Muller et al. 2015). Due to their bi-directional activity, AtUMAMITs are involved in multiple physiological roles ranging from phloem loading/unloading, over xylem-phloem transport, to transport to sink tissues (Ladwig et al. 2012; Muller et al. 2015; Besnard et al. 2016). When looking at the cell layer-specific transcript abundance, all differentially regulated AtUMAMIT genes found upon presence of the hemibiotroph *P. parasitica* were downregulated: *AtUMAMIT11/38/41* were differentially regulated in the rhizodermis and the cortex. Besides, *AtUMAMIT11* was additionally downregulated in the stele. *AtUMAMIT33* was regulated in the cortex and *AtUMAMIT5* in the rhizodermis as well as the stele (Froschel et al. 2021). *AtUMAMIT18* expression in the rhizodermis and stele, *AtUMAMIT5* in the stele, and *AtUMAMIT34* expression in the cortex were downregulated upon hemibiotrophic *V. longisporum* infection. The opposite effect, an increase in transcripts, was seen for *AtUMAMIT5/31* (cortex), *AtUMAMIT38* (endodermis) and *AtUMAMIT14* (stele) (Froschel et al. 2021). Based on the analysis of transgenic *Arabidopsis* lines, Besnard et al. (2021) suggested that AtUMAMIT14 is a positive regulator in plant pathogen resistance. When challenged with the biotrophic oomycota *Hyaloperonospora arabidopsidis*, *AtUMAMIT14* overexpression lines displayed enhanced expression of SA marker genes as well as SA levels, leading to increased resistance (Besnard et al. 2021). The example of UMAMITs visualizes a diverse set of responses, where individual genes can be regulated opposingly depending on the cell type, and genes within the transporter family are regulated inconsistently. It might be that their bi-directional transport ability causes different responses, which is why the individual role of each transporter during plant-pathogen interaction needs to be carefully evaluated.

### Lysine histidine transporters (LHTs)

In *Arabidopsis*, 10 AtLHT paralogs (Rentsch et al. 2007) exist with different specificity and cellular location. AtLHT1, the first identified transporter of this family (Chen and Bush 1997; Hirner et al. 2006; Svennerstam et al. 2007) is involved in leaf mesophyll import as well as root uptake of acidic and neutral AAs, both at naturally occurring concentrations (Svennerstam et al. 2011), and from agricultural soil (Ganeteg et al. 2017). AtLHT1 also transports non-proteinogenic AAs, like 1-aminocyclopropane-1-carboxylic acid (ACC), just as its paralog AtLHT2 (Shin et al. 2015; Choi et al. 2019). ACC serves as a precursor of the phytohormone ethylene (ET) and as a signaling molecule on its own (Van de Poel and Van Der Straeten 2014; Vanderstraeten et al. 2019). AtLHT1 can be exploited to shuttle novel AA-coupled pesticides inside a plant (Jiang et al. 2018; Chen et al. 2018). Homologs of AtLHT1 were also identified and studied in, e.g., rice, poplar, lotus, tea and ginseng (Guether et al. 2011; Zhang et al. 2013; Wang et al. 2019; Guo et al. 2020; Gratz et al. 2021; Li et al. 2021). The *Arabidopsis* knockout mutant *lht1-1* displayed an early senescence phenotype (Hirner et al. 2006; Svennerstam et al. 2007).

The role of AtLHT1 during pathogen infection has been investigated in several studies: *AtLHT1* transcript levels were elevated when the host was infected with the biotrophic powdery mildew fungus *Erysiphe cichoracearum* (*E. cichoracearum*) (Liu et al. 2010) or the biotrophic nematode *Heterodera schachtii* (Elashry et al. 2013). Also, upon infection with the hemibiotrophic bacteria *P. syringae*, the fungi *Colletotrichum higginsianum* (*C. higginsianum*) (Liu et al. 2010) and *V. longisporum* (Froschel et al. 2021) as well as the oomycete *P. parasitica* (Froschel et al. 2021), *AtLHT1* was upregulated. Most biotrophs feed on the apoplast or apoplast-like compartments and assimilate nutrients directly from their living host (Szabo and Bushnell 2001; Fatima and Senthil-Kumar 2015; Wang et al. 2020). It has been shown, that pathogens can reprogram plant transport proteins for their benefit, in order to, e.g., gain nutrients (Delmotte et al. 2009; Spanu and Panstruga 2017). This opens for the possibility that the pathogen, rather than the host plant, may steer the expression of *AtLHT1*.

**Fig. 1:**
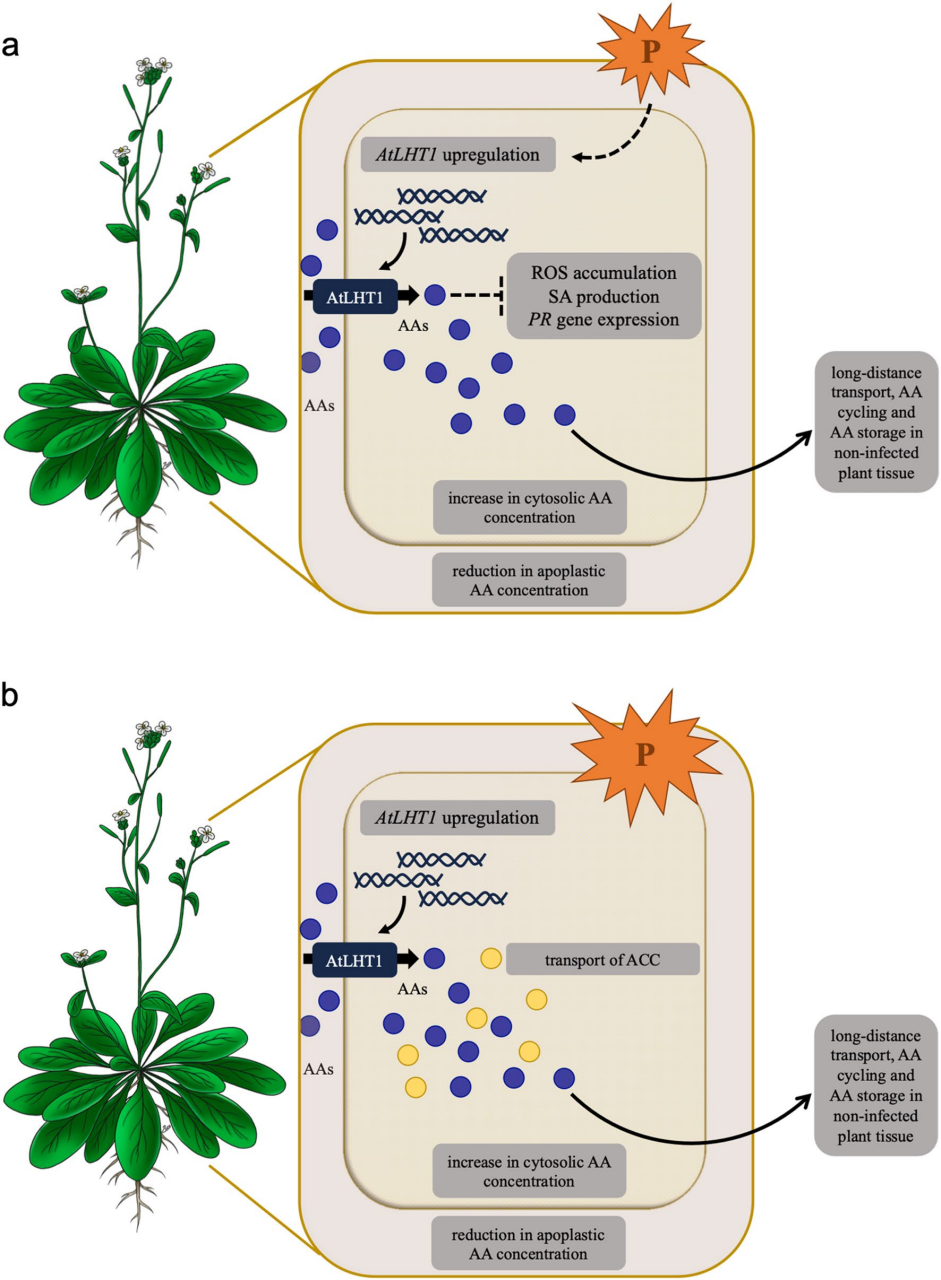
Response of the plant amino acid transporter AtLHT1 to pathogen attack. Upon attack by biotrophic pathogens (orange P), the transcript abundance of *AtLHT1* is increased (**a**). An increased gene expression leads to an increased AtLHT1 protein abundance at the plasma membrane, which causes an active import of AAs (purple dots) into the cytosol. As a consequence, a depletion of apoplastic- and an increase of cytosolic AA concentrations occurs. This might be a direct response by the plant to apoplastic-feeding pathogens, in order to empty the apoplast and shuttle AAs into the cytosol. From there, AAs can be exported to healthy plant tissues. Due to the fact that *lht1-1* mutants display increased pathogen resistence due to the accumulation of reactive oxygen species (ROS), salicylic acid (SA) production and pathogenesis-related (PR) gene expression, the upregulation of *AtLHT1* might by steered by the biotrophic pathogen itself (dotted arrow). This action might avoid SA defense responses and might increase chances for the pathogen to survive. Upon attack by a necrotrophic pathogen, *AtLHT1* is also elevated (**b**). This might, however, be an exclusive response by the plant. AtLHT1 transports the ethylene precursor 1-aminocyclopropane-1-carboxylic acid (ACC) (yellow dots). Mostly ET/JA-mediated responses contribute to the defense against necrotrophic pathogens. Additionally, an upregulation of the transporter might contribute to the shuttling of AAs to healthy, more distal plant tissues. Hence, the observed upregulation of *AtLHT1* might be mostly a protective measure, steered by the plant

From a plant’s perspective, it would be beneficial to increase the uptake of AAs from the apoplast to lower AAs accessibility for biotrophic pathogens and to secure its AA resources away from the infected area. This means an increased remobilization of AAs would require increased expression of AATs as part of a slash-and-burn defense strategy (Masclaux-Daubresse et al. 2010) (Fig. 1a). The increased expression of *AtLHT1* could be seen as a defense strategy caused by the plant to drain a maximum of AAs out of the apoplast in order to starve the pathogen.

However, and in contrast to the predictions from this hypothesis, *lht1-1* knockout mutants displayed increased resistance to *P. syringae, C. higginsianum* and *E. cichoracearum*, highlighting that AtLHT1 is a negative regulator in plant defenses (Liu et al. 2010). Disruption of *AtLHT1* displayed different defense responses such as increased callose deposition, hypersensitive cell death and the constitutive expression of genes belonging to the SA defense pathway such as *PR1* (Liu et al. 2010). The response is very similar to what was described for mutants of different AAPs (Elashry et al. 2013; Marella et al. 2013; Berg et al. 2021; Froschel et al. 2021). Liu et al. (2010) hypothesized that AtLHT1’s role in plant resistance was linked to its ability to transport glutamine. The absence of AtLHT1 causes a lack of glutamine within the cell, which leads to an altered redox status and enhanced immunity due to an accumulation of reactive oxygen species (ROS) and induced programmed cell death (PCD) (Liu et al. 2010). This suggests that the increased expression of *AtLHT1* observed during the infection may be caused by the biotrophic pathogens, in order to inhibit the activation of the SA defense and, hence, an increase in plant resistance (Fig. 1a).

On the contrary, necrotrophic pathogens break plasma membranes and induce PCD in the host prior to nutrient uptake. While the SA pathway plays little role, the ethylene/jasmonic acid (ET/JA)-mediated response contributes to defense against necrotrophic pathogens (Glazebrook 2005; Pieterse et al. 2012; Huang et al. 2020). Furthermore, it has been shown that plants react in an analogous way to nematodes as to necrotrophic pathogens by activating the ET/JA pathway (Przybylska and Obrępalska-Stęplowska 2020)*.* Similar to what has been observed for biotrophic pathogens, increased *LHT1* transcript levels were also found upon interaction with necrotrophic pathogens *Botrytis cinerea* (Xiong et al. 2018) and *Erwinia amylovora* (Farjad et al. 2018). Farjad et al. confirmed the involvement of *AtLHT1* during pathogen attack: *AtLHT1* resembled the expression profile of other defense associated genes by being induced during infection, behaving opposing to other N metabolism related genes. Potentially this serves an increased transport of ACC, supporting ET-based plant defense, as AtLHT1 and AtLHT2 were found to transport the ET precursor (Shin et al. 2015; Choi et al. 2019). This hypothesis is in line with the finding, that *lht1-1* mutants displayed no increased resistance to necrotrophic pathogen infection such as *Sclerotinia sclerotiorum* (Liu et al. 2010) or the nematode *H. schachtii* (Elashry et al. 2013). Necrotrophic pathogens would not benefit from increasing the transcript abundance of *AtLHT1*, which therefore might display a plant response in order to transport ACC as defense mechanism as well as to transport AAs away from the invaded tissue (Fig. 1b).

## Regulation of amino acid transporters through additional physiological processes

**Fig. 2:**
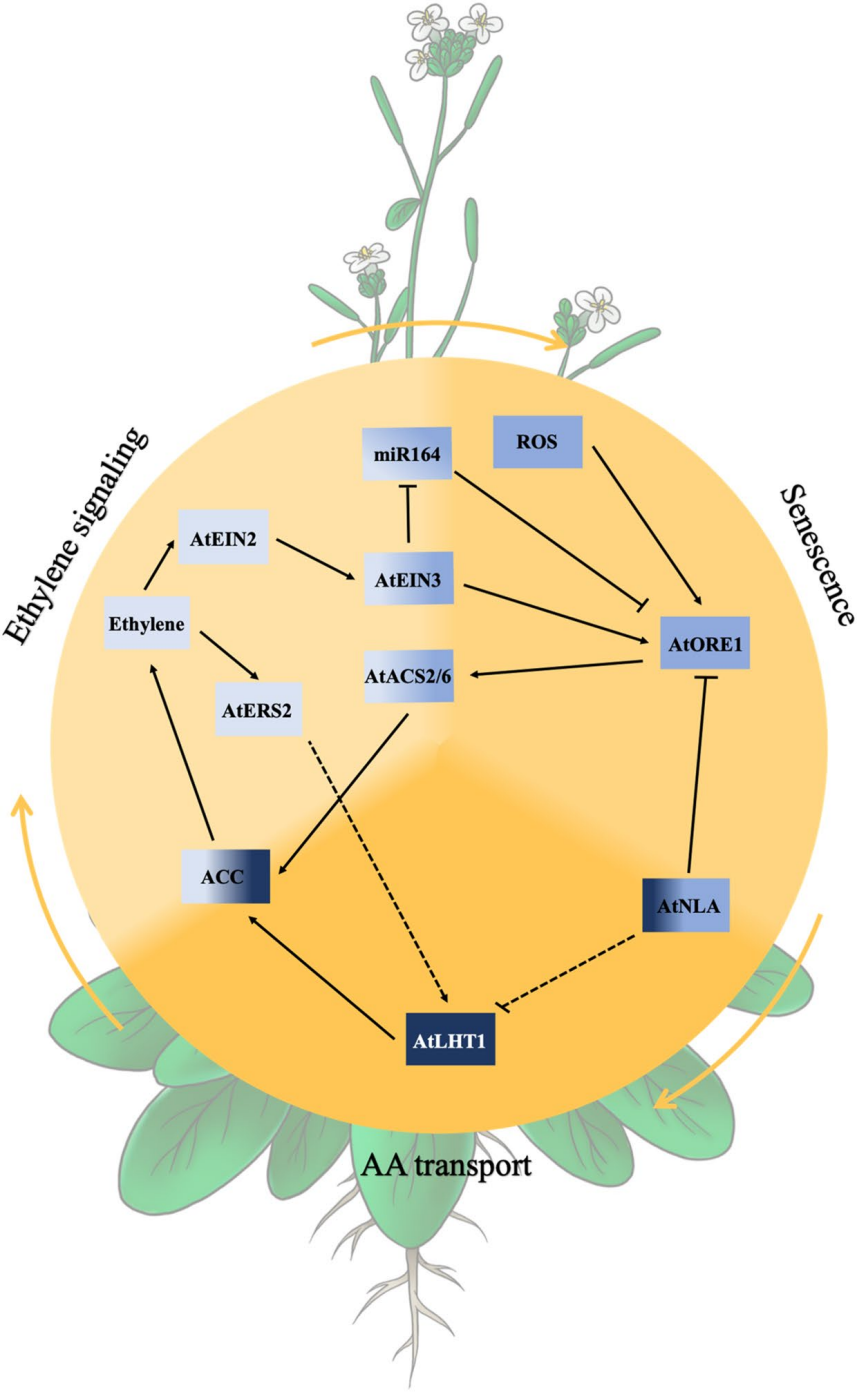
The molecular regulation of amino acid transporters is influenced by diverse regulatory pathways. Using the example of AtLHT1, the influence of individual key players important for ethylene (ET) signaling and senesence in the context of pathogen defense is depicted. AtLHT1 transports the signaling molecule and ET precursor 1-aminocyclopropane-1-carboxylic acid (ACC). The ET receptor kinase ETHYLENE RESPONSE SENSOR2 (AtERS2) might interact with AtLHT1 and thus depicts a potential feedback loop in dependence of ET. ET presence in parallel represses the activity of miRNA164, through the action of the transcription factor (TF) EIN3. miRNA164 itself is a negative regulator of the TF ORESARA1 (AtORE1), a key player in plant senescence. AtEIN3 activates *AtORE1* transcription directly whereas AtORE1 then activates the expression of *ACC SYNTHASE2* (*AtACS2*), displaying a feed-forward loop. AtORE1 itself is directly regulated by the ubiquitin ligase NITROGEN LIMITATION ADAPTATION (AtNLA), which also regulates AtLHT1 through either direct or indirect action. Dashed lines indicate potential regulatory connections that remains to be tested

The dominant players in plant defense are the antagonistic phytohormones SA and ET/JA (Huang et al. 2020; Zhang et al. 2020). The involvement of other phytohormones and crosstalk among the different players is well studied (Pieterse et al. 2012; Huang et al. 2020; Zhang et al. 2020; Aerts et al. 2021). The SA-mediated defense seems to be more effective against biotrophs and hemibiotrophs whereat the ET/JA-mediated defense targets necrotrophic microbes (Glazebrook 2005; Huang et al. 2020; Zhang et al. 2020). The link between SA-mediated defense and AAT regulation has been studied (Liu et al. 2010; Yang et al. 2014; Besnard et al. 2021), whereas not much is known about ET/JA-regulated defense against necrotrophs in connection to AAT regulation. Recently, much work has been done on understanding the molecular underpinnings of leaf senescence. Due to the fact that the *lht1-1* mutant displays an early senescence-like phenotype (Hirner et al. 2006; Svennerstam et al. 2007), we aimed to identify regulatory targets, that play a role in plant senescence and pathogen defense, and at the same time display a connection to the regulation of AATs (Fig. 2).

The transcription factor ORESARA1 (AtORE1) targets promoters of senescence-associated genes and directly mediates PCD (Oh et al. 1997; Kim et al. 2009; Balazadeh et al. 2010; Farage-Barhom et al. 2011; Al-Daoud and Cameron 2011; Matallana-Ramirez et al. 2013; Qiu et al. 2015; Durian et al. 2020). AtORE1 itself is targeted for degradation by the RING-type E3 ubiquitin ligase NITROGEN LIMITATION ADAPTATION (AtNLA) (Park et al. 2018). Deubiquitination events, however, stabilize AtORE1 and promote leaf senescence (Park et al. 2019). ET is involved in a positive regulation of *AtORE1.* More specifically, AtEIN3, a transcription factor acting downstream of EIN2, represses *miR164*, a negative regulator of *AtORE1*, and can in parallel bind to the *AtORE1* promoter (Kim et al. 2009, 2014; Li et al. 2013). Together with AtEIN3, *AtORE1* then activates transcription of chlorophyll catabolic genes in an ET dependent manner (Qiu et al. 2015). AtORE1 additionally activates *ACC SYNTHASE2* (*AtACS2*) and *AtACS6* expression, leading to enhanced ET production, displaying a coherent feed-forward loop for ET dependent leaf senescence (Qiu et al. 2015; Zhang et al. 2021). Interestingly, the action of AtORE1 and AtNLA are tightly connected to plant defense responses (Zhang et al. 2021). *Arabidopsis* infection with the hemibiotroph *V. dahliae* caused premature leaf senescence. It was shown that a microbial elicitor interfered with the interaction between AtORE1 and AtNLA, which, in turn, stabilized AtORE1, enhanced ET production and, thus, promoted senescence (Zhang et al. 2021). Recently, it was shown that AtORE1 is activated through protein phosphorylation via the calcium (Ca^2+^) kinase AtCPK1 (Durian et al. 2020). This kinase has previously been analyzed and it was shown that *AtCPK1* is upregulated upon pathogen infection and was found to be a positive regulator in plant resistance due to activation of SA biosynthesis (Coca and San Segundo 2010). Interestingly, also plants infected with necrotrophs displayed increased resistance, although no ET derived defense responses were found (Coca and San Segundo 2010). In a preprinted study, it was suggested that AtNLA displays a negative regulator in plant defense against necrotrophs (Val-Torregrosa et al. 2021-preprint). *nla* mutants displayed increased callose deposition as well as increased resistance. Upon pathogen attack, transcript levels of *AtNLA* were reduced (Val-Torregrosa et al. 2021-preprint).

It was recently shown that AtORE1 and AtNLA additionally play a role in the regulation of AtLHT1 (Fig. 2). The ubiquitin ligase AtNLA targets pathways connected to organic N remobilization by targeting AATs during N deficiency (Liao et al. 2020). Transcript abundance of several AATs was found upregulated in the *nla* mutant and *AtLHT1* displayed the highest regulation. A proteomic analysis confirmed the regulation of AtLHT1 by AtNLA (Liao et al. 2020), however, it remains to be tested whether this regulation is due to a direct interaction between AtLHT1 and the ligase. The authors additionally speculated whether AtORE1 is controlling transcription of *AtLHT1* (Liao et al. 2020), however, an upregulation of *AtLHT1* in *AtORE1* overexpression lines has not been observed (Matallana-Ramirez et al. 2013). It remains unclear whether AtORE1 serves as TF regulating *AtLHT1*.

Given this complex regulatory crosstalk between different physiological processes, it can be speculated whether AtLHT1 is subject to additional molecular regulation. Due to the fact that miR164 is an important player at the interface between ET signaling and senescence (Kim et al. 2009, 2014; Li et al. 2013), and miRNAs in general play important roles in plant immunity (Val-Torregrosa et al. 2021), future studies should evaluate whether *AtLHT1* may also be regulated through the action of miRNAs. As mentioned above, the signaling compound and ET precursor ACC is transported by members of the AtLHT family (Van de Poel and Van Der Straeten 2014; Shin et al. 2015; Choi et al. 2019; Vanderstraeten et al. 2019), which provides a direct link between the ET signaling- and AA uptake pathways. In addition, Chen et al 2012 found the ER-localized ETHYLENE RESPONSE SENSOR2 (ERS2) (Hua et al. 1998), to interact with AtLHT1 in yeast (Chen et al. 2012). Novel findings about the poplar homolog PtrLHT1.2 being not exclusively localized at the PM but also at the ER (Gratz et al. 2021), raise the question about a potential functional importance of this potential interaction, that remains to be tested *in planta*. Given the fact that ERS2 is a receptor kinase (Moussatche and Klee 2004) whose activity is not needed for ET signaling, it raises the question whether the kinase targets substrates outside the ET pathway and, thus, could be involved in additional responses (Chen et al. 2009; Lacey and Binder 2014). This opens up for the hypothesis that AtLHT1 could be post-translationally modified in an ET-dependent way; a speculation that remains to be tested. The strong connection between AtLHT1 and ET leads to the question if unknown defense responses against necrotrophs exist, that involve the action of AtLHT1. Pathogen attack triggers Ca^2+^ influx into the cell (Nishad et al. 2020), which can then lead to phosphorylation and activation of AtORE1 (Coca and San Segundo 2010; Durian et al. 2020). Overexpression of *AtCPK1* leads to increased resistance of plants upon necrotrophic attack, the molecular regulation for this is, however, so far unknown (Coca and San Segundo 2010). The suggested downregulation of *AtNLA* upon necrotrophic interaction (Val-Torregrosa et al. 2021-preprint) would lead to a potential reduction in AtORE1 degradation. Overall, this would increase AtORE1 activity and PCD as well as senescence (Oh et al. 1997; Kim et al. 2009; Balazadeh et al. 2010; Farage-Barhom et al. 2011; Al-Daoud and Cameron 2011; Matallana-Ramirez et al. 2013; Qiu et al. 2015; Durian et al. 2020). This, a beneficial outcome for necrotrophs, would stand in contrast to the fact that a high accumulation of AtORE1 would increase ACC production via ACS2/6, and thus, ET accumulation (Qiu et al. 2015; Zhang et al. 2021). Reduced transcript accumulation of *AtNLA* would additionally lead to an increase in *AtLHT1* (Liao et al. 2020). AtLHT1 could then contribute to the production of ET by transport of ACC (Shin et al. 2015; Choi et al. 2019) and, potentially, ET triggered resistance to necrotrophic microbes. It becomes evident that many common players in the regulation of pathogen resistance, leaf senescence and AAT regulation have overlapping functions. In future experiments, it has to be carefully determined, in which way the crosstalk between those players has an influence on plant microbes and plant resistance.

The complex network behind plant pathogen defense depends on several factors such as soil N availability and composition of the soil N pool which would affect both the internal N status of the plant and its energy status. Both plants and pathogens possess toolboxes, containing different signaling molecules such as ROS or hormones, but also transcription factors to concur the respective other. These responses are deeply interwoven with a machinery of cell-type specific regulation of AATs and, hence, the accumulation or depletion of specific AAs. The unique response signatures that are being formed upon association of a pathogen then contributes to the susceptibility of the plant.

## Supplementary Information

Below is the link to the electronic supplementary material.

Supplementary file1 (XLSX 24 kb)

Supplementary file2 (DOCX 21 kb)

## Data Availability

Not applicable.
