# Supplementary material for: To have or not to have: expression of amino acid transporters during pathogen infection
Source: Plant Mol Biol. 2022 Feb 1;109(4-5):413–25. doi: 10.1007/s11103-022-01244-1 (PMC9213295; doi:10.1007/s11103-022-01244-1)
Supplement: Supplementary file 2 — Supplementary file2 (DOCX 21 kb) [file 11103_2022_1244_MOESM2_ESM.docx]

**References Supplementary Tables 1-3**

Agrios G (2005) Environmental effects on the development of infectious plant disease. Plant Pathology 5:249-264. doi:10.1016/B978-0-08-047378-9.50013-0

Chi WJ, Wang ZY, Liu JM, Zhang C, Wu YH, Bai YJ (2019) Ammonium uptake and assimilation are required for rice defense against sheath blight disease. Cereal Research Communications 47 (1):98-110. doi:10.1556/0806.46.2018.056

Ding S, Shao X, Li J, Ahammed GJ, Yao Y, Ding J, Hu Z, Yu J, Shi K (2021) Nitrogen forms and metabolism affect plant defence to foliar and root pathogens in tomato. Plant Cell Environ 44 (5):1596-1610. doi:10.1111/pce.14019

Duffy B, Défago G (1999) Macro-and microelement fertilizers influence the severity of Fusarium crown and root rot of tomato in a soilless production system. HortScience 34 (2):287-291. doi:https://doi.org/10.21273/HORTSCI.34.2.287

Elmer WH (1997) Influence of chloride and nitrogen form on Rhizoctonia root and crown rot of table beets. Plant disease 81 (6):635-640. doi:https://doi.org/10.1094/PDIS.1997.81.6.635

Elmer WH, LaMondia JA (1999) Influence of Ammonium Sulfate and Rotation Crops on Strawberry Black Root Rot. Plant Dis 83 (2):119-123. doi:10.1094/PDIS.1999.83.2.119

Farjad M, Rigault M, Pateyron S, Martin-Magniette ML, Krapp A, Meyer C, Fagard M (2018) Nitrogen Limitation Alters the Response of Specific Genes to Biotic Stress. Int J Mol Sci 19 (11):3364. doi:10.3390/ijms19113364

Fernandez-Crespo E, Scalschi L, Llorens E, Garcia-Agustin P, Camanes G (2015) NH4+ protects tomato plants against Pseudomonas syringae by activation of systemic acquired acclimation. J Exp Bot 66 (21):6777-6790. doi:10.1093/jxb/erv382

Gupta KJ, Brotman Y, Segu S, Zeier T, Zeier J, Persijn ST, Cristescu SM, Harren FJ, Bauwe H, Fernie AR, Kaiser WM, Mur LA (2013) The form of nitrogen nutrition affects resistance against Pseudomonas syringae pv. phaseolicola in tobacco. J Exp Bot 64 (2):553-568. doi:10.1093/jxb/ers348

Harrison UJ, Shew H (2001) Effects of soil pH and nitrogen fertility on the population dynamics of Thielaviopsis basicola. Plant and Soil 228 (2):147-155. doi:doi.org/10.1023/A:1004845715885

Hoffland E, Jeger MJ, van Beusichem ML (2000) Effect of nitrogen supply rate on disease resistance in tomato depends on the pathogen. Plant and Soil 218 (1):239-247. doi:doi.org/10.1023/A:1014960507981

Hoffland E, van Beusichem ML, Jeger MJ (1999) Nitrogen availability and susceptibility of tomato leaves to Botrytis cinerea. Plant and Soil 210 (2):263-272

Huber D, Watson R (1974) Nitrogen form and plant disease. Annual review of phytopathology 12 (1):139-165. doi:doi.org/10.1146/annurev.py.12.090174.001035

Lecompte F, Abro MA, Nicot PC (2010) Contrasted responses of Botrytis cinerea isolates developing on tomato plants grown under different nitrogen nutrition regimes. Plant pathology 59 (5):891-899. doi:https://doi.org/10.1111/j.1365-3059.2010.02320.x

Soulie MC, Koka SM, Floch K, Vancostenoble B, Barbe D, Daviere A, Soubigou‐Taconnat L, Brunaud V, Poussereau N, Loisel E (2020) Plant nitrogen supply affects the Botrytis cinerea infection process and modulates known and novel virulence factors. Mol Plant Pathol 21 (11):1436-1450. doi:doi/pdfdirect/10.1111/mpp.12984

Wang M, Sun Y, Gu Z, Wang R, Sun G, Zhu C, Guo S, Shen Q (2016) Nitrate Protects Cucumber Plants Against Fusarium oxysporum by Regulating Citrate Exudation. Plant Cell Physiol 57 (9):2001-2012. doi:10.1093/pcp/pcw124

Zhou J, Wang M, Sun Y, Gu Z, Wang R, Saydin A, Shen Q, Guo S (2017) Nitrate increased cucumber tolerance to Fusarium wilt by regulating fungal toxin production and distribution. Toxins 9 (3):100. doi:DOI:10.3390/toxins9030100
